# Curved Fabry-Pérot Ultrasound Detectors: Optical and Mechanical Analysis

**DOI:** 10.3390/s25041014

**Published:** 2025-02-08

**Authors:** Barbara Rossi, Maria Alessandra Cutolo, Martino Giaquinto, Andrea Cusano, Giovanni Breglio

**Affiliations:** 1 Department of Electrical Engineering and Information Technology, University of Naples Federico II, 80138 Napoli, Italy; breglio@unina.it; 2 Optoelectronic Division, Engineering Department, University of Sannio, 82100 Benevento, Italy; acusano@unisannio.it; 3 Department of Information Engineering, Electrical Engineering and Applied Mathematics, University of Salerno, 84084 Fisciano, Italy; mgiaquinto@unisa.it

**Keywords:** optoacoustic detector, Fabry–Perot interferometer, lab-on-fiber technology, fiber optic, fiber optics sensors, polymeric micro-structure

## Abstract

Optical fiber-based acoustic detectors for ultrasound imaging in medical field feature plano-concave Fabry–Perot cavities integrated on fiber tips, realized via dip-coating. This technique imposes constraints on sensor geometry, potentially limiting performance. Lab-on-Fiber technology enables complex three-dimensional structures with precise control over geometric parameters, such as the curvature radius. A careful investigation of the optical and mechanical aspects involved in the sensors’ performances is crucial for determining the design rules of such probes. In this study, we numerically analyzed the impact of curvature on the optical and acoustic properties of a plano-concave cavity using the Finite Element Method. Performance metrics, including sensitivity, bandwidth, and directivity, were compared to planar Fabry–Perot configurations. The results suggest that introducing curvature significantly enhances sensitivity by improving light confinement, especially for cavity thicknesses exceeding half the Rayleigh zone (∼45 μm), reaching an enhancement of 2.5 a *L* = 60 μm compared to planar designs. The curved structure maintains high spectral quality (FOM) despite 2% fabrication perturbations. A mechanical analysis confirms no disadvantages in acoustic response and bandwidth (∼40 MHz). These findings establish curved plano-concave structures as robust and reliable for high-sensitivity polymeric lab-on-fiber ultrasound detectors, offering improved performance and fabrication tolerance for MHz-scale bandwidth applications.

## 1. Introduction

The advancement of lab-on-fiber (LOF) technology has led to the development of minimally invasive optical probes. LOF allows the functionalization of an optical fiber through the integration of micro- and nanostructures on the fiber tip [1,2,3]. These optical probes find widespread applications in the medical field, thanks to their biocompatibility, compact size, lack of electrical connection, and great sensitivity. Specifically, in ultrasound imaging, there is increasing interest in the realization of minimally invasive devices [4,5]. The sensitive detection of broadband ultrasound waves ranging from hundreds of kHz to tens of MHz is essential for techniques like biomedical photoacoustic and clinical ultrasound high-resolution imaging [6]. Traditionally, piezoelectric transducers (PZTs) are employed for both the generation and detection of ultrasound [7,8]. Small piezoelectric probes present a challenge in terms of high reception sensitivity and broadband transmission. In this context, an all-optical ultrasound-based device offers a valid alternative to piezoelectric technology. All-optical ultrasound-based systems usually consist of two optical fibers, one for the detection and one for the generation of ultrasound waves [9]. The generation system exploits the photoacoustic effect [10]. The optical fiber delivers light to a target of interest, where it is optically excited and absorbed via specific tissue before being rapidly converted to heat, resulting in a slight temperature increase. This causes an initial pressure increase, which then relaxes, resulting in the emission of broadband (about tens of megahertz) low-amplitude (less than 10 kPa) acoustic waves [6,11]. The acoustic waves are then detected via an optical fiber-based acoustic detector, which typically exploits interferometric structures integrated on the fiber tip [12,13,14]. Fabry–Perot (FP) interferometers are commonly used for acoustic detection, with length variations caused by an incident acoustic field modulating the interferometric fringes occurring in the fiber reflectivity spectrum [15]. However, they suffer from a drop in performance due to the divergent effect of the beam [16,17]. To address this problem, Guggenheim et al. [18] proposed a curved FP that allows for refocusing the light, thereby resolving the optical problem of the planar FP solution and becoming the state-of-the-art acoustic detector. Curved structures realized on the tip of optical fiber are already implemented in all-optical ultrasound systems for imaging applications. For example, S. Zhang et al. proposed a fiber-optic system capable of simultaneous laser interstitial thermal therapy (LITT) and real-time in situ all-optical ultrasound imaging for lesion monitoring [19]. Their devices included three optical fibers: one for ultrasound transmission and reception and another for providing thermal therapy light. Furthermore, Z. Zhang et al. developed a catheter designed specifically for side-viewing B-mode ultrasound imaging of the upper gastrointestinal tract [4]. X. Guo et al. realized an all-optical fiber ultrasound probe for temperature measurement during radiofrequency ablation [20]. Curved ultrasound detectors have usually been developed utilizing the dip-coating technique [21]. Significant limitations stem from the inherent difficulties in fabrication, where geometrical constraints and manufacturing tolerances have to be taken into account. For instance, the accessible surface area for those realized cavities is naturally confined by the fiber’s diameter, which restricts the amount of optical material deposition (typically through the dip coating technique), resulting in a lack of fine control over the radius of curvature. Furthermore, manufacturing tolerances are crucial since even minor errors in the fabrication process can significantly impact the final device’s performance and reproducibility. Consequently, the curvature may not fully correct the divergence of the desired light beam, potentially resulting in inferior performance in applications requiring precise control over light propagation. New fabrication processes, such as the Two-Photon Polymerization (2PP) technique [22], allow for more precise structural realization [23,24]. This advancement enables the design of structures capable of fully correcting beam divergence. In this work, we thoroughly analyze the Curved FP cavity by numerically evaluating its optical and mechanical behavior by means of a Finite Element Method-based model. Throughout the study, the results are compared to a benchmark configuration provided by a “classic” planar surface FP cavity to better understand the influence of the introduction of curvature. We also assess the tolerance for deviations from ideal conditions, such as curvature due to manufacturing procedures. Finally, we evaluate the structure’s sensitivity and bandwidth for three different sensor cavity lengths in response to an incident acoustic wave.

## 2. Methods and Metrics

The acoustic ultrasound fiber-based detector analyzed in this work is composed of a polymeric interferometric cavity integrated into the optical fiber tip to give rise to interference fringes in the fiber reflection spectrum (typically spectral dips). An incident acoustic wave of amplitude (P) can deform the cavity, thus causing a variation in its length (L). Therefore, this cavity works as a sensing element, basically relying on the detection of the spectral dip shift caused by the incident acoustic field. Given the small entity (a few picometers) and the high frequencies (up to the MHz range), the shift is typically detected with a tunable laser set to a specific wavelength, and the signal can be monitored with a circulator and a photodiode  [19].

### 2.1. Aim of the Carried-Out-Analysis

**Figure 1:**
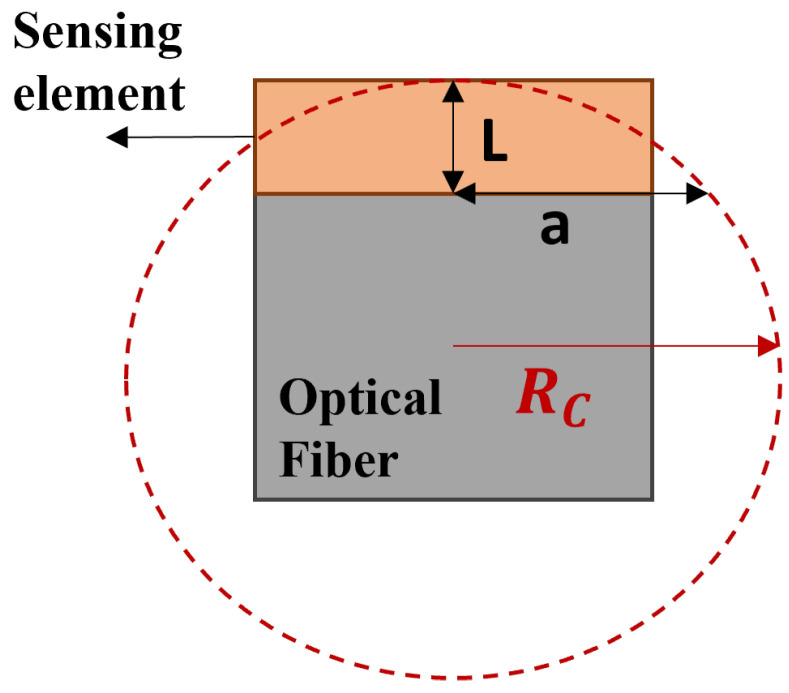
Schematic representation of the model with sensitive element (orange) on the optical fiber tip (gray).

Plano-concave configurations have demonstrated considerable benefits in terms of performance, [16,21,25], mainly from an experimental point of view. However, understanding the impact of a curved surface on the previously introduced optical and acoustic key parameters can be crucial for defining the sensitivity during the design phase, according to the degrees of freedom offered through modern fabrication approaches. Specifically, we conducted a comparative study between the curved configuration and a planar FP interferometer under similar conditions, including Gaussian beam propagation and an incident acoustic wave input. As a theoretical benchmark, in our analysis we also considered the optical response of an ideal FP system simulated as an infinitely extended FP cavity (labeled Ideal FP) excited via a plane wave with a normal incidence. As depicted in the schematic of Figure 1, the curved surface FP cavity was modeled as a spherical shell with a cavity length *L* and a radius of curvature as follows:

(1) $$L=R_{C}\pm \sqrt{R_{C}^{2}-a^{2}}$$

where a is the base radius of the spherical shell, actually matching that of the fiber tip cross-section. Note that these characteristics are limited by geometric constraints imposed by the structure’s fabrication on fiber, such as fiber diameter, polymer volume, surface tension, fiber tip diameter, and cavity length [26]. Accordingly, with Equation (1), typically used dip coating approaches lead to a reduced cavity length with an increase in the fiber diameter. However, modern technologies, for example, those based on two-photon polymerization (2PP), allow the realization of configurations with a sphero-cylinder shape, thus overcoming this trade-off and offering more degrees of freedom for optimizing the performances. This aspect can be crucial, especially considering that the mechanical behavior, which also defines the frequency bandwidth, is strongly related to the effective thickness of the structure. Moreover, assuming that the dependability and effectiveness of sensor systems are heavily reliant on obtaining repeatability in mass production settings and maintaining resilience against potential response variances, we also included an analysis concerning the influence of geometrical tolerances on performance. As stated before, a curved FP structure is typically achieved using the dip coating technique [24], where factors such as the volume of polymer used, surface tension, fiber tip diameter, and cavity length strongly influence the curvature, making precise control over the curvature radius challenging. Conversely, a planar FP structure is typically realized through the spin coating technique [27], though achieving flawlessly parallel structures on optical fiber tips also remains a challenging task.

### 2.2. Metrics’ Definitions

The sensitivity *S* of the sensors can be described as the variation in the reflectivity spectrum ΔR as a function of the incident field amplitude (*P*) at a fixed optical working wavelength λ, as outlined in Equation (2) [28,29]:

(2) $$S={\left.\frac{dR}{dP}\right|}_{\lambda =\bar{\lambda }}={\left.\frac{dR}{d\lambda }\right|}_{\lambda =\bar{\lambda }}\;\cdot \;\frac{d\lambda }{dL}\;\cdot \;\frac{dL}{dP}=S_{R}\;\cdot \;S_{\lambda }\;\cdot \;S_{A}$$

where *R* is the reflection spectrum. When a linear behavior is assumed, the sensitivity can be, in principle, expressed as a product of two factors: one related to the mechanical response (cavity length variation ΔL as a function of acoustic wave amplitude *P*), hereinafter defined a “acoustic sensitivity”, $S_{A}$, and one related to the optical response, i.e., the “optical sensitivity”, $S_{O}$, that describes the reflectivity spectrum change ΔR at a specific wavelength, $\bar{\lambda }$, as a function of cavity length variation. Moreover, the optical sensitivity, $S_{O}$, can in turn be expressed as the product of two parameters: $S_{R}={\left.\frac{dR}{d\lambda }\right|}_{\lambda =\bar{\lambda }}$, which is the spectral dip slope at the working wavelength, and $S_{\lambda }=\frac{d\lambda }{dL}$, which is the dip wavelength shift induced via the cavity length variation. As a result, the sensitivity *S* is determined by three important parameters: $S_{R}$ and $S_{\lambda }$, which are related to optical behavior, and $S_{A}$, which pertains to the acoustic response.

It is worth observing that the first key parameter, $S_{R}$, is related to the spectral dip shape: the sharper the dip, the higher this sensitivity component. Therefore, it is essential to maximize both the quality factor (*Q*) and visibility (*V*) of the spectral dip. *Q* is defined as the ratio between the wavelength λ corresponding to the minimum reflection dip position and the dip width evaluated as the full width at the half maximum (FWHM), as expressed in (3):

(3) $$Q=\frac{\lambda_{\min}}{\mathrm{FWHM}},$$

*V* is the dip amplitude, evaluated accordingly with (4) as the difference between the reflectivity baseline $R_{\mathrm{MAX}}$ and the minimum value assumed in correspondence of the spectral dip $R_{\mathrm{MIN}}$:

(4) $$V=R_{\mathrm{MAX}}-R_{\mathrm{MIN}}.$$

The figure of merit (FOM) is given by the product of *Q* and *V*:

(5) FOM=V×Q

In our analysis, we will also consider the FOM as a compact and concise parameter given by the product of *Q* and *V* (Equation (5)). The second parameter contributing to the sensitivity, $S_{\lambda }$, characterizes the device’s ability to convert thickness variations into a measurable optical parameter, in accordance with the FP theory. The third parameter, $S_{A}=\frac{dL}{dP}$, is determined by the displacement distribution across the cavity caused by the incident acoustic field. The displacement is strongly dependent on the acoustic frequency. It is important to consider that, for photoacoustic imaging, where such a miniaturized sensor finds its main application and utility, working bandwidths of tens of MHz are required [30].

### 2.3. Numerical Model Adopted

**Figure 2:**
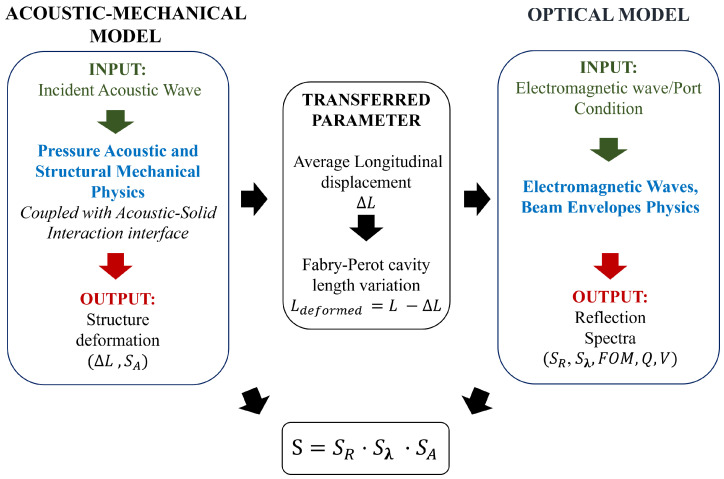
Diagram of FEM models in Comsol Multphysics, illustrating the interaction between the acoustic–mechanical and optical models used. The acoustic–mechanical model, solved using Pressure Acoustics and Structural Mechanics physics in COMSOL coupled with the Acoustic-Solid Interaction interface, simulates the structural response to an incident acoustic wave. The acoustic–mechanical model’s output, representing the polymer’s deformation (ΔL), serves as the initial condition for the optical analysis, affecting the cavity thickness ($L_{\mathrm{Deformed}}=L-\Delta L$) and reflection spectrum. The optical model, using the Electromagnetic Wave, Beam Envelopes Physics module, computes the reflection spectra and optical sensitivity. The overall sensitivity of the sensor is determined by combining the acoustic and optical sensitivities.

In our analysis, we assumed that the sensing element is illuminated by a Gaussian beam with a beam radius of 5.8 μm [31]. A Gaussian beam can be modeled considering the following expression:

(6) $$w\left(z\right)=w_{0}\sqrt{1+{\left(\frac{z}{Z_{R}}\right)}^{2}}$$

(7) $$Z_{R}=\frac{\pi w_{0}^{2}}{\lambda }$$

(8) $$R_{beam}\left(z\right)=z\left[1+{\left(\frac{\pi w_{0}^{2}}{z\lambda }\right)}^{2}\right]$$

where *w* is the beam waist, $Z_{R}$ is the Rayleigh zone, and $R_{beam}$ is the radius of curvature of the beam at a certain abscissa *z* parallel to the light’s propagation direction, and $w_{0}$ is the beam waist at the abscissa z=0 assumed at the end of the optical fiber. According to Equations (6) and (7), the Rayleigh zone obtained is approximately 90 μm. The specific curvature radii $R_{c}$ used in the following analysis were selected to numerically match the radius of curvature of the Gaussian beam $R_{beam}$ at a specific axial position calculated using Equation (8). This choice ensures optimal coupling and interaction between the beam and the structure with an increased sensitivity. The analysis was conducted with a Finite Element Method approach using the commercial Comsol Multiphysics, a software produced by *COMSOL AB*, Tegnérgatan 23, SE-111 40 Stockholm, Sweden. As previously highlighted, the analysis was carried out from both acoustic and optical standpoints. A schematic diagram of the model is presented in Figure 2.

The acoustic analysis was carried out using a Multiphysics simulation in COMSOL. Following a method proposed in previous works, it is addressed using an acoustic–mechanical model that requires the use of two physics interfaces: Pressure Acoustics and Structural Mechanics, as described in our previous works  [28]. The propagation of the acoustic wave along the longitudinal direction of the model is essentially ruled by the Helmholtz equation  [32]. To properly couple these physics, it is necessary to impose the continuity of normal displacement at the solid–fluid interfaces, ensure static equilibrium between the pressure and the stress normal to the solid boundaries, and enforce zero tangential stresses at the fluid boundaries  [33]. The external domain was surrounded by water, and impedance boundary conditions were applied at its boundaries to minimize artificial reflections and ensure that the simulation accurately represents an infinite domain. The dimension of the external domain was chosen in such a way as to place the boundary domain far enough from the sensor to not influence the computed solution and small enough to not affect the computational cost of the simulation. The polymeric structure was modeled using a linear elastic and isotropic material  [34], and the fiber was modeled with the “rigid boundary” condition taking into account the higher Young modulus of the fiber (70 GPa) compared with that of the polymer. The optical model, carried out with the Electromagnetic Wave, Beam Envelopes Physics module, evaluates the reflection spectra parameters and the optical sensitivity. The geometry is surrounded by Perfectly Matched Layers to absorb any outgoing waves. To compute how much of the light is guided in the fiber, we used a port of the numeric type in the model. The Boundary Mode Analysis study steps computed the eigenmodes and propagation constants of the fibers. The final frequency domain study step solves the electric field in the domains and the S-parameters. The acoustic–mechanical and optical models were coupled using a compact parameter as a quantifier of the polymer deformation under acoustic excitation, which, in turn, affects the interaction between the polymeric cavity and the light emitted from the fiber. In more detail, in the optical model, the compact parameter affects the cavity thickness, leading to a change in the reflection spectrum. Based on these considerations, the solution obtained using the acoustic–mechanical model was used as the initial condition for the optical analysis, as better explained in the “Sensitivity evaluation” section. Finally, the product of the optical sensitivity and acoustic sensitivity allows the determination of the overall sensitivity behavior of the sensors as a function of acoustic frequency.

Regarding the mesh, two distinct configurations were tailored and optimized to address the specific requirements of the optical and acoustic–mechanical interaction problems, taking into account the different wavelengths involved. This approach provided a good balance between computational efficiency and simulation accuracy. In the optical model, a mapped mesh was employed, with refinement concentrated in the sensor area, where the light is most intensely focused. For the acoustic–mechanical model, a free tetrahedral mesh was utilized, with refinement applied near the sensor and in regions exhibiting significant displacement. In the surrounding water domain, the mesh size was determined by the frequency, f, of the acoustic wave, using the equation 1500/f/10, where 1500 m/s represents the assumed speed of sound in water, well approximating that of soft tissues.

Without a loss of generality, we consider a polymeric curved surface cavity with a refractive index of n = 1.52 [35]. We also included thin gold layers at the top (i.e., on the polymeric structure) and the bottom (i.e., on the fiber tip) to improve the reflectivity at the edge cavity [36]. The structure was immersed in a water domain (n = 1.33). Additionally, we utilized a single-mode optical fiber with a core diameter of 8 μm and a core refractive index (n) of 1.46  [37]. The optical working wavelength range considered in this study is between 1.4 μm and 1.6 μm, which is the typical range adopted with standard optical telecom instrumentation. This range, in fact, corresponds to the region of minimized loss in the spectrum of single-mode optical fiber. Tailoring the design to the operating range of commercial single-mode optical fibers would be beneficial in future works oriented to the concrete realization and test of the analyzed devices. We simulated a polymeric resin material known as IP-Dip, part of the IP Photoresins optimized for 2PP [22] produced by *Nanoscribe GmbH & Co. KG*, Germany (info@nanoscribe.com). These resins were chosen due to their exceptional properties, which are critical for high-precision additive manufacturing. IP-Dip resins are particularly renowned for enabling the fabrication of complex microstructures with outstanding detail and accuracy, making them ideal for applications requiring precise geometries. Furthermore, their biocompatibility makes them suitable for biomedical and biological applications, where interaction with living systems is a key consideration. Importantly, the refractive index and mechanical properties of IP-Dip align with the specific requirements of our design, ensuring optimal optical performance and structural stability  [38]. These characteristics, combined with the flexibility offered by 2PP, also guided the selection of curvature radii to achieve a numerical match with the Gaussian beam at a specific axial position. According to [39], Ip-Dip had a Young’s Modulus of E=2.75GPa, a Poisson ratio ν=0.35, and a density $\rho =1.14\;{g/\mathrm{cm}}^{3}$. The proposed structures are thought for photoacoustic and/or ultrasound imaging applications, which require a sensitivity that allows the detection of pressure amplitudes in the kPa range [6,18,24].

## 3. Numerical Analysis

### 3.1. Optical Spectral Analysis

**Figure 3:**
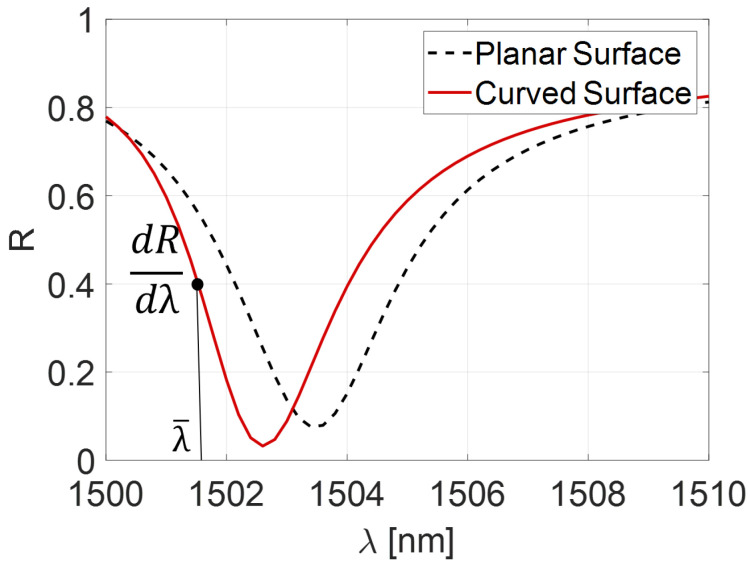
Reflectivity spectrum of curved surface FP (red solid line) and planar surface (black dashed line) for a 45 µm cavity length.

This section investigates the effect of curvature on the optical spectral characteristics of an FP cavity. The results obtained with a Curved FP were compared to a Planar FP of the same cavity thickness and simulated under the same conditions. In Figure 3, we present a reflection spectrum obtained with an FP cavity of 45 μm thickness (half of the Rayleigh zone). It is worth noting that the spectrum of the Planar FP degrades, but curvature reduces this deterioration, as expected. Moreover, we conducted a parametric analysis as a function of the cavity thickness. Equation (1) demonstrates a trade-off between fiber size and effective film thickness. Indeed, increasing the fiber size allows for obtaining a reduced cavity length. It is also important to underline that, in the design of optical sensors for photoacoustic imaging applications, it is critical to find a compromise between sensitivity and bandwidth. Specifically, an intrinsic trade-off exists between cavity length and bandwidth: increasing the cavity thickness reduces the latter [29]. For this reason, a range of 10 μm to 80 μm is considered in the following analysis, as will be clarified in the next section.

**Figure 4:**
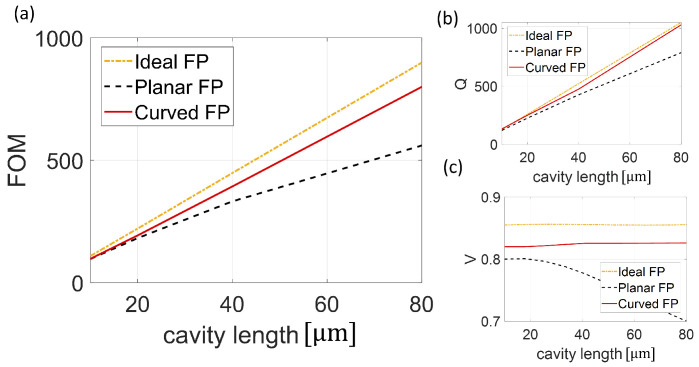
(**a**–**c**) Optical parameters FOM, Q-factor, and Visibility of the Ideal Fabry Perot (yellow dash and dot line), Planar FP (black dash line), and Curved FP (red solid line).

**Figure 5:**
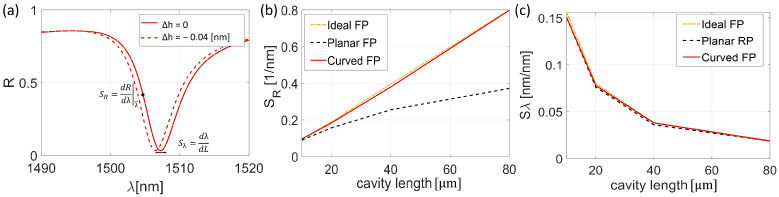
(**a**) Reflection Spectra evolution of Curved FP as a function of the cavity length variation around a nominal value of 20 µm. (**b**) $S_{R}$ and (**c**) Sλ as a function of the cavity length of the Ideal Fabry Perot (yellow dashed point line), Planar Fabry Perot (black dashed line), and Curved FP (red solid line).

As discussed in the previous section, the spectral shape plays a fundamental role in enhancing device performance. The trends of parameters FOM, Q-factor, and Visibility (V) (Figure 4), as well as $S_{R}$ and $S_{\lambda }$ (Figure 5) as functions of cavity length, were analyzed. Figure 4a shows the FOM of the three different configurations, namely Ideal FP, Planar FP, and Curved FP. Note that the Curved FP follows the behavior of the Ideal FP with a slope of 0.010 and 0.011, respectively, while the Planar FP configuration has a reduced FOM, which notably worsens beyond 40 μm with a slope of 0.0038. In more detail, the Q-factor of all three configurations increases with the cavity length (Figure 4b). Specifically, the Ideal FP and Curved FP had a slope of 0.0132 and 0.0130, respectively, whereas the Planar FP had a lower slope of 0.0095. The parameter that most affected the FOM is V, shown in Figure 4c. The V of the curved surface configuration is almost constant with a value of 83% throughout the whole investigated cavity length, only 2% less than in the ideal case. On the other hand, the Planar FP *V* starts with a value of 0.8, with a marked degradation from 20 μm to 80 μm with a slope of 0.027, reaching a value of 0.7 at 80 μm.

Figure 5 schematically depicts the previously defined $S_{R}$ and $S_{\lambda }$ parameters for a Curved FP cavity length of 20 μm with a variation of ΔL=0.04, as will be clarified later. The trends of $S_{R}$ and $S_{\lambda }$ parameters are presented in Figure 5b,c as a function of cavity length. It is worth noting that the falling edge slope of the interference peak at the working wavelength $S_{R}$ has different results for the Curved FP and Planar FP (Figure 5b). Indeed, the Curved FP structure follows the Ideal FP trend with a slope of 0.010, while the Planar FP suffers a steady drop in $S_{R}$, starting with a slower slope of 0.005 up to 40 μm and reduced to a slope of 0.003.

On the other hand, all three models showed a comparable shift in the peak interference dip, $S_{\lambda }$, which followed a decreasing exponential trend when the cavity thickness was varied (Figure 5c). It is possible to observe that the parameters $S_{\lambda }$ and $S_{R}$ have an opposite trend as a function of the cavity length. More specifically, a cavity length reduction also causes a broadening of the resonant dip, compensated with a higher wavelength shift induced via the perturbation ΔL. The results suggest that $S_{R}$ is the parameter most affected by the increase in cavity thickness, particularly as the beam begins to diverge. Overall, the optical spectral analysis reveals that the curved structure’s optical behavior improves when the cavity width increases, especially in terms of $S_{R}$ and FOM, heading toward the theoretical ideal performances, whereas the Planar FP structure degrades. Note that, for thicknesses below 20 μm, introducing a curved surface does not appear to introduce significant advantages in terms of sensitivity. However, at 45 μm (half of the Rayleigh zone), the planar structure’s performance deteriorates, while introducing curvature improves the optical response.

### 3.2. Impact of Non-Idealities on the Spectral Shape

A curved FP is typically realized using the dip coating technique [17,18]. In this process, the curvature is influenced by various factors, including the volume of polymer used, surface tension, fiber tip diameter, and cavity length. Consequently, it is challenging to maintain perfect control over the curvature radius. On the other hand, Planar FP is usually fabricated through the spin coating technique [27], and achieving a flawlessly parallel FP structure on the tip of an optical fiber remains a difficult task. In the realm of sensor manufacturing, achieving consistency across large quantities is paramount. This consistency ensures that every sensor performs reliably within the specified parameters, regardless of production scale. However, it is equally important to design these systems to be resilient to slight variations that might occur during the manufacturing process. When dealing with sensor arrays, particularly in multiplexed configurations, even minor deviations in fabrication can significantly affect the overall performance. These variations can introduce discrepancies in the optical properties of the sensors, leading to inconsistent data and unreliable performance [40]. In this section, we examined the fabrication tolerance of both sensors. In more detail, we analyzed the reflection spectra variation due to a perturbation of the ideal value identified for the curved and planar FP of a 20 μm cavity length, capable of performing high-frequency signal detection [21,36].

**Figure 6:**
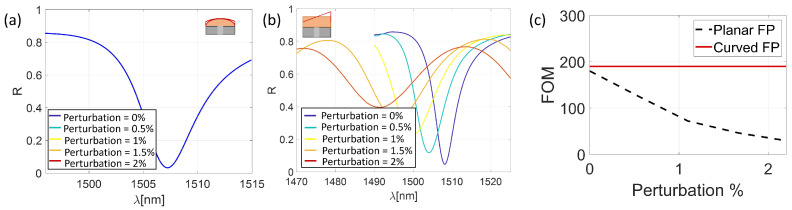
(**a**) Reflection spectra of a curved FP as a function of radius of curvature perturbation. (**b**) Reflection spectra of a planar FP as a function of the variation in the inclination of the upper plane. (**c**) FOM as a function of the perturbation applied to the optimum condition of a planar FP (black dashed line) and a curved FP (red solid line).

Figure 6a shows the interference spectra of the curved FP with an $R_{C}$ percentage variation of up to 2% from the established optimal value (i.e., the radius of curvature matched with the curvature of the Gaussian beam front). The spectral dip is unaffected by radius perturbation, demonstrating the curved structure’s robustness. Interestingly, the reflection spectrum is invariant to changes in the radius of curvature. To assess the tolerance of a planar FP, we calculated the reflectivity as a function of the perturbation of the FP’s top surface tilting (inset of Figure 6b). The spectra in Figure 6b show that a small perturbation, less than 2%, resulted in a drastic reduction in performance. In order to quantify these aspects, in Figure 6c, we plot the FOM as a function of the perturbation for both the analyzed configurations. The FOM actually provides a simple description of the discussed spectral degradation, capturing essential properties like *V* and peak sharpness, which are critical for evaluating sensor performance under different settings. Note that the flatness of a Planar FP is critical to maintaining a high FOM. A tiny 2% fluctuation within fabrication tolerances can dramatically reduce the FOM by one order of magnitude, from 180 to 29. Conversely, a disturbance of the same magnitude in the curvature radius does not affect the curved-surface FP’s spectrum. The reliability and effectiveness of sensor systems rely heavily on ensuring repeatability in mass production settings, as well as resistance to potential response variations. As a result, the curved FP, although not manifesting advantages in terms of sensitivity at low thicknesses, provides a more robust and tolerant solution.

### 3.3. Acoustic Analysis

**Figure 7:**
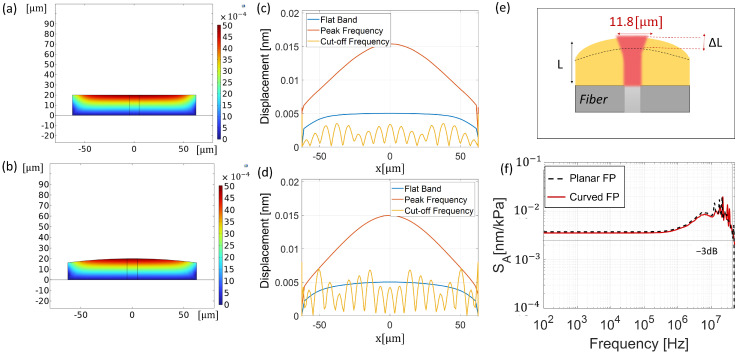
Longitudinal displacement field maps evaluated in the correspondence of a flat band in the case of (**a**) the planar FP and (**b**) the curved FP. Longitudinal displacement for three different acoustic wave frequencies: flat band (1 kHz) (blue solid line), peak frequency in the bandwidth (13 MHz) (orange solid line), and cut-off frequency (42 MHz) (yellow solid line) of the planar FP (**c**). Longitudinal displacement for three different acoustic wave frequencies: flat band (1 kHz) (blue solid line), peak frequency in the bandwidth (14 MHz) (orange solid line), and cut-off frequency (41 MHz) (yellow solid line) of the curved FP (**d**). (**e**) Schematic representation of average displacement ΔL calculated in the region of interest irradiated with light (11.8 µm). (**f**) Longitudinal displacement of the area illuminated by the beam (5.8 µm) as a function of frequency for the planar FP (black dashed line) and curved FP (red solid line).

We investigated the displacement distribution along the sensor showing the longitudinal displacement field maps of the planar and curved FP structures at 1 kHz (Figure 7a and Figure 7b, respectively). The field maps reveal that displacement is concentrated near the center of the structure in both configurations, while the edges are actually unchanged. This aspect is also highlighted in Figure 7c,d,where the longitudinal displacement distribution at 1 kHz is represented as a function of the spatial coordinate x orthogonal to the fiber longitudinal axis (blue curve). Figure 7c,d also represent the longitudinal displacement distribution at different frequencies, cut-off frequency, and peak frequency, parameters that will be thereafter discussed. Accordingly, with these results, from the displacement maps, we evaluated the maximum longitudinal displacement ΔL (corresponding to that evaluated in the central region) as a synthetic and explainable parameter characterizing the mechanical behavior of the analyzed structures. The central region also overlaps with the ”active area” of our sensor Figure 7e, reasonably identified by the region illuminated by the Gaussian beam exiting from the fiber core (whose extension is defined by Equation (6)). Therefore, ΔL was evaluated as an average value across the active area, which for a cavity length of 20 μm has an extension radius *w* of 11.8 μm. The average operation allows for extracting a reliable synthetic parameter even in the case of strong displacement fluctuations occurring at high-frequency ranges (see yellow curves in Figure 7c,d. Following this approach, it is possible to evaluate the frequency response of the analyzed configurations.

**Figure 8:**
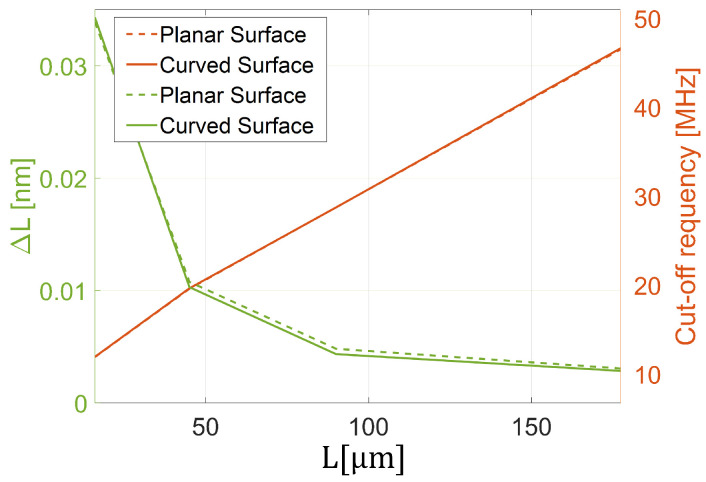
Displacement and cut-off frequency as a function of the cavity length.

Indeed, in Figure 7f, we show the acoustic sensitivity, $S_{A}$, as a function of the impinging acoustic field frequency. The results suggest that the curved FP exhibits an acoustic behavior (red solid curve) that is essentially the same as that obtained for a planar configuration (black dashed curve). The sensitivity is flat over a wide frequency range, assuming a value of $S_{A}=0.004\;\left[\mathrm{nm}/\mathrm{kPa}\right]$ with an overshoot of $S_{A}=0.0169\;\left[\mathrm{nm}/\mathrm{kPa}\right]$ at the peak frequency of 12 MHz. This overshoot, according to the results shown in Figure 7c,d (orange curves), is due to an increased displacement concentration in the central region, at the expense of the areas outside the active one. At higher frequencies, the sensitivity decreases, reaching a cut-off frequency (evaluated at —3 dB) of 41 MHz, at which the displacement appears to be not uniformly distributed within the central active region (see the yellow curves in Figure 7c,d), due to the instantiation of higher-order vibrational modes. The central displacement, ΔL, has, then, been analyzed as a function of the cavity length, *L*, obtaining the trends plotted in Figure 8 (green curve). Consistently with the results discussed so far, the curved configuration ΔL exhibits the same trend (solid green curve) obtained with the planar configuration (dashed green curve). The results also overlap in terms of cut-off frequency, which has an approximately linear increase as a function of the cavity length increase. Actually, the curved configuration suffers from the same trade-off between acoustic sensitivity and bandwidth characterizing the flat FP configurations [18] (Figure 8).

**Figure 9:**
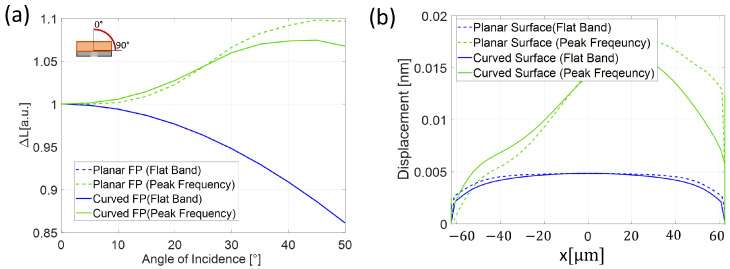
(**a**) Longitudinal displacement as a function of the angle of incidence. (**b**) Longitudinal displacement along the planar (dashed line) and curved (solid line) FP in the flat band and peak frequency.

To conclude, we also evaluated the directional response of the proposed structures both in the flat band and at peak frequency. Figure 9a shows ΔL (normalized for visualization convenience) as a function of the angle of incidence in the flat band (blue) and at the peak frequency (green). In more detail, there is an overlap between the two ΔL as a function of the angle of incidence at lower frequencies. Note also that there is a trade-off between displacement and the angle of incidence: increasing the angle of incidence decreases the displacement. The flat band displacement reaches, in both cases, a reduction of 15% for an angle of incidence of 50 degrees. At peak frequency, the curved FP has an almost constant displacement up to an angle of 20° and then increases linearly with a slope of 0.0026 between 20° and 30°, reaching a plateau between 30° and 50°. On the other hand, the planar FP increases with a steeper slope of 0.0027 from 10° up to 45°. Additionally, we considered how the longitudinal displacement was distributed along the structure, i.e., as a function of the spatial coordinate of the structures (*x*) in the flat band and at peak frequency (Figure 9b). An average value of the 25° angle of incidence corresponding to the linear response zone of both ΔL configurations was considered. In Figure 9b, while both structures exhibit a displacement mainly concentrated at the center within the flat band, increasing the frequency resulted in different displacement distributions, which vary, depending on the angle of incidence and the geometry of the structures. Overall, the curved FP appears to have a more stable response to the angle of incidence variation.

## 4. Sensitivity Evaluation

**Figure 10:**
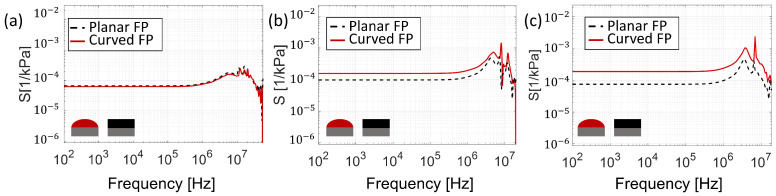
Sensitivity as a function of the acoustic frequency of a curved surface and a planar surface for different cavity lengths, 20 μm (**a**), 45 μm (**b**), and 60 μm (**c**).

As explained in the metrics section, the *S*, i.e., the reflectivity variation caused by the pressure of an incident acoustic wave at an operative wavelength, $\bar{\lambda }$ (Equation (2)), can be decomposed into three key parameters. We thoroughly investigated each of them, $S_{R}$, $S_{\lambda }$, and $S_{A}$, to determine the optimization margins for each parameter. As a result, we can recompose these insights to calculate sensitivity. To accomplish this, we must relate the structure’s deformation, as determined by the acoustic analysis, to the optical analysis in a simple, computationally efficient, and, most importantly, explainable method. The acoustic analysis revealed that the displacement is mainly concentrated in the structure’s center, with the edges remaining relatively steady. It is also worth noting that the optical fiber’s light is concentrated in the sensor’s center zone; therefore, the displacement parameter we choose corresponds to the region lighted by the fiber, which contributes greatly to the sensor’s sensitivity. For this reason, we defined a compact parameter, ΔL, which served as the starting point for the optical analysis. This quantity represents the average displacement in the cavity’s center. Using this information, we performed an optical analysis that coherently incorporates the mechanical deformation. In more detail, the displacement (ΔL) was applied centrally on the cavity length, *L*, of the curved construction, resulting in a new cavity length, ($L_{\mathrm{Deformed}}=L-\Delta L$). This correction also changed the radius of curvature, which affected the structure’s optical response. This allows us to determine the sensor’s sensitivity and bandwidth dependent on the frequency of the incident acoustic wave. This aspect is relevant in the field of photoacoustic imaging (PAI) since having a wide bandwidth is essential for PAI applications because it provides higher resolution and more detailed data. Figure 10 shows the sensitivity *S* as a function of frequency for three different cavity lengths suitable for photoacoustic imaging and/or ultrasound imaging (20 μm, 45 μm, and 60 μm).

The performances of the proposed structures are reported in Table 1. The sensitivity for a curved FP of 20 μm (Figure 10a) is $S_{c}=6.26\times 10^{-5}\;\left[{\mathrm{kPa}}^{-1}\right]$ with a cut-off frequency of $f_{c}=41\mathrm{MHz}$, while the planar FP configuration has a sensitivity $S_{p}=6.61\times 10^{-5}\;\left[{\mathrm{kPa}}^{-1}\right]$ with a cut-off frequency of $f_{p}=42\mathrm{MHz}$. As the cavity thickness increases, the divergent character of the beam improves, enhancing the effect of curvature (Figure 10b,c). At 45 μm, we can observe a variation of an order of magnitude, ranging from $S_{p}=9.67\times 10^{-5}\;\left[{\mathrm{kPa}}^{-1}\right]$ to $S_{c}=1.56\times 10^{-4}\;\left[{\mathrm{kPa}}^{-1}\right]$ of the curved FP (Figure 10b) with a bandwidth of 14.6 MHz and 14.0 MHz, respectively. Finally, Figure 10c shows the sensitivity response for a cavity length of 60 μm. The sensitivity of the curved surface is $S_{c}=1.88\times 10^{-4}\;\left[{\mathrm{kPa}}^{-1}\right]$ with a cut-off frequency $f_{c}=6.4\mathrm{MHz}$. Note that the curved FP reaches an enhancing factor of 2.5 compared with the planar FP. Furthermore, as the cavity thickness increases, the sensitivity of the planar FP exhibits a contrasting trend compared to the curved counterpart. Indeed, while the curved FP has an improvement in sensitivity with an increasing thickness, in the planar configuration, there is a reduction in sensitivity at 60 μm due to the worsening of optical behavior, as highlighted in the optical spectral analysis.

## 5. Conclusions

In this paper, we have presented a numerical analysis of a curved surface FP structure integrated on an optical fiber tip for ultrasound detection, focusing on sensor performance in terms of sensitivity S, bandwidth, directivity, and fabrication tolerance. Using Finite Element Method (FEM) approaches, we compared the impact of curvature on the optical and mechanical aspects of the FP structure with a planar FP simulated under the same conditions.

The results indicate that the curved FP sharpens the spectral dip, enhancing the sensor’s optical sensitivity. Furthermore, it significantly improves the sensor’s robustness, maintaining a stable figure of merit (FOM) even with structural perturbations of around 2%, demonstrating superior resilience to fabrication errors. The mechanical analysis demonstrates that the introduction of curvature does not adversely impact the acoustic response, particularly in terms of bandwidth. Furthermore, the curved FP structure exhibits enhanced stability in response to variations in the angle of incidence.

Overall, the curved FP structure offers improved repeatability, fabrication tolerance, and higher sensitivity due to better optical field confinement, especially for cavity thicknesses exceeding half the Rayleigh zone 45 μm, achieving an enhancement factor in S of 2.5 at L = 60 μm. As such, it is a promising candidate for high-sensitivity polymer-based lab-on-fiber ultrasound detectors designed for MHz-scale bandwidth applications.

Although experimental validation has not yet been conducted for the proposed structures, prior studies have demonstrated the feasibility of fabricating complex 3D microstructures directly on optical fiber tips using the 2PP technique [23,24]. These examples underscore the versatility and reliability of 2PP in creating functional microstructures, even on challenging substrates like optical fibers. These prior studies not only validate the technical feasibility of fabricating similar structures, allowing for future experimental efforts to translate the proposed designs into practical devices, but also pave the way for the design (and consequent realization and testing) of more complex geometries. In fact, advances in 2PP technology continue to enhance its resolution and material compatibility, ensuring the precise control of parameters such as the curvature radius, as discussed in this study. Moreover, the biocompatibility of IP photoresins used in 2PP further broadens the potential for integrating these structures into biomedical applications. Together, these advancements lay a solid groundwork for the development of high-performance, polymer-based lab-on-fiber ultrasound detectors, offering significant potential for the future of medical diagnostics and sensing technologies.

## Figures and Tables

**Table 1:** Performances of the proposed structures.

Cavity Length	Configuration	Sensitivity	Cut-Off Frequency
**[μm]**		**[kPa^−1^]**	**[MHz]**
20	Curved FP	$6.26\times 10^{-5}$	41
	Planar FP	$6.61\times 10^{-5}$	42
45	Curved FP	$15.6\times 10^{-5}$	14.6
	Planar FP	$9.67\times 10^{-5}$	14.0
60	Curved FP	$18.8\times 10^{-5}$	6.4
	Planar FP	$7.50\times 10^{-5}$	6.0

## Data Availability

Data is contained within the article. For further information or data requests, the authors are available and can be contacted.
